# Supplementary material for: Doxycycline for the prevention of progression of COVID-19 to severe disease requiring intensive care unit (ICU) admission: A randomized, controlled, open-label, parallel group trial (DOXPREVENT.ICU)
Source: PLoS One. 2023 Jan 23;18(1):e0280745. doi: 10.1371/journal.pone.0280745 (PMC9870104; doi:10.1371/journal.pone.0280745)
Supplement: S1 Table — (DOCX) [file pone.0280745.s001.docx]

**Supplementary Table 1.** **Number of Risk factors associated with severe COVID-19 in the study.** Absolute numbers of Risk factors (RF; comorbidities associated with the risk for developing severe COVID-19) in patients randomized to SoC and SoC+Doxy groups.

| **RF (n)** | **Randomised to SoC+Doxy (n=192)** | | **Randomised to SoC (n=195)** | |
| --- | --- | --- | --- | --- |
|  | **n with RF** | **RF total (n)** | **n with RF** | **RF total (n)** |
| 0 | 48 | 0 | 49 | 0 |
| 2 | 3 | 6 | 7 | 14 |
| 3 | 44 | 132 | 44 | 132 |
| 4 | 55 | 220 | 52 | 208 |
| 5 | 40 | 200 | 34 | 170 |
| 6 | 2 | 12 | 8 | 48 |
| 7 | 0 | 0 | 1 | 7 |
|  | | **Total RF (n) = 570** |  | **Total RF (n) = 579** |
|  |  | average/all = 3.0 |  | average/all = 3.0 |
|  |  | average/RF+ = 4.0 |  | average/RF+ = 4.0 |
