# Supplementary figures and images for: Doxycycline for the prevention of progression of COVID-19 to severe disease requiring intensive care unit (ICU) admission: A randomized, controlled, open-label, parallel group trial (DOXPREVENT.ICU)
Source: PLoS One. 2023 Jan 23;18(1):e0280745. doi: 10.1371/journal.pone.0280745 (PMC9870104; doi:10.1371/journal.pone.0280745)

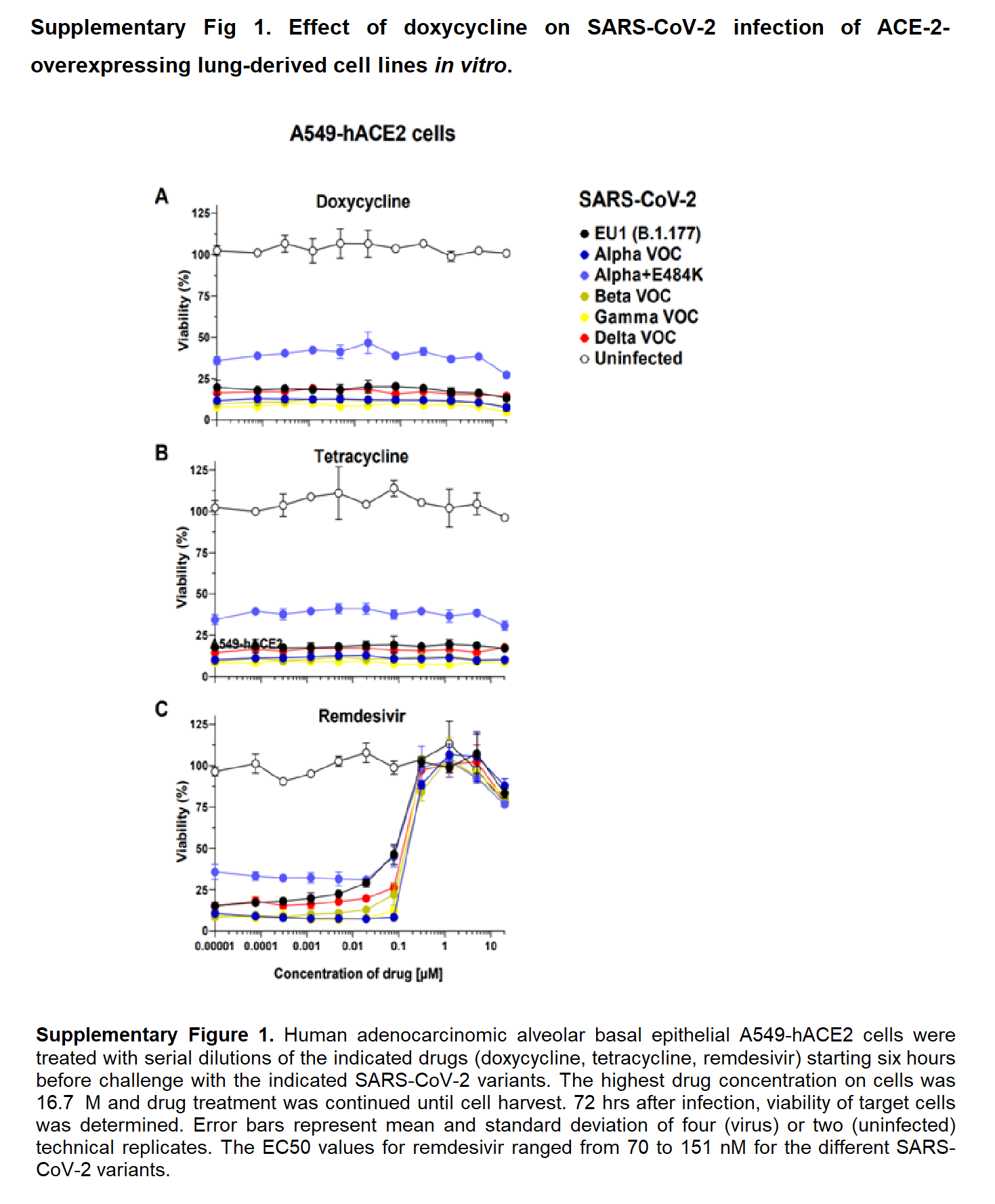

Supplement: S1 Fig — Effect of doxycycline and tetracycline compared to remdesivir on SARS-COV-2 infection of ACE-2-overexpressing lung-derived cell lines in vitro. (TIF) [file pone.0280745.s002.tif]
