# Supplementary material for: Doxycycline for the prevention of progression of COVID-19 to severe disease requiring intensive care unit (ICU) admission: A randomized, controlled, open-label, parallel group trial (DOXPREVENT.ICU)
Source: PLoS One. 2023 Jan 23;18(1):e0280745. doi: 10.1371/journal.pone.0280745 (PMC9870104; doi:10.1371/journal.pone.0280745)
Supplement: S1 File — (PDF) [file pone.0280745.s003.pdf]

Oral DOxycline for the PREVENTion of severe COVID-19 ICU Admission (DOXPREVENT.ICU)

Stephan D. Gadola, MD, PhD, FRCP, John Kirkpatrick, MSc, Raja Dhar, MD, Ratko Djukanovic, MD, DM, FRCP

Abstract: COVID-19 progresses to severe and critical illness in 14% and 6% of SARS-CoV-2 infected patients, respectively. Here, we propose a pragmatic clinical trial to test oral doxycycline as a therapy for newly hospitalized COVID-19 patients who are at high risk for progression to severe disease, with the main objective to reduce the burden of critical care (CC) services resulting from admission of severe patients in CC facilities.
The choice of doxycycline is based on its broad antimicrobial efficacy, including antiviral properties; recent in silico analyses identifying it as a potential strong inhibitor of NSP5, a key pathogenic protein of SARS-CoV-2; its targeted anti-inflammatory properties and efficacy in human virus-induced hyperinflammation (in dengue fever); as well as its excellent safety profile, pharmacokinetic properties, low price, and its world-wide availability due to its status a one of the essential medicines defined by the WHO.

A pragmatic controlled trial with hard clinical endpoints is proposed, which should produce short-term results to enable rapid implementation of the new therapeutic strategy if successful.

I. Study Rationale

<u>I.I. Unmet need for preventative strategies to reduce the number of severe COVID-19 cases:</u>

According to the recent update from the ECDC "based on the predicted development of the 14-day cumulative notification rate, similar levels (of COVID-19) to those seen in Hubei providence are expected to be seen in all EU/EEA countries and the UK in a few days to a few weeks." (1). Therefore, there is a very high risk that the ICU capacity of the health care systems in the UK and other European countries will soon be exceeded.

Current clinical trials, such as Genentech's Actemra trial, or the adaptive platform trial REMAP-CAP focus on already severely ill COVID-19 patients. Indeed, patients need to be already admitted to an area that is designated for critical care to be eligible for REMAP-CAP. These trials will therefore not provide sufficiently comprehensive strategies to prevent progression to severe disease. Furthermore, the primary endpoint of the REMAP-CAP trial is all-cause mortality at 90 days, which means that implementation of the results of this large trial may be difficult to implement in a timely manner to influence treatment policies during the current pandemic.

In the current situation, pragmatic controlled trial designs that can be easily applied to the real-life situation in a busy hospital ward should be given strong consideration. Here, we propose a proof-of-concept trial with a 14-days treatment period, robust clinical endpoints, and short follow-up. If successful, the trial could be easily expanded to gain further scientific insights, while starting implementation of the new strategy in parallel.

<u>I.II. Rationale for testing doxycycline in COVID-19:</u>

1. Broad antimicrobial activity, including anti-viral activity: Doxycycline is efficacious against a broad spectrum of bacteria and other pathogens, including intracellular plasmodia (2), chlamydia (3), and rickettsia (4), and various viruses. Indeed, it has been shown to exert potent anti-viral activity *in vitro* and *in vivo* against dengue virus (5-7), chikungunya (8), vesicular stomatitis virus (9), and others (e.g. 10). At a standard dose of 200mg/d doxycycline reduced mortality in Dengue fever patients by 46%, in a placebo-controlled, randomized trial in 216 patients (6). Of note, Covid-19 patients can be misdiagnosed for Dengue due to false positive serological Dengue test results, indicating substantial structural similarities of the two viruses (11).

2. Potential direct antiviral activity of doxycycline against SARS-CoV-2: In a new study using high throughput virtual ligand screening on SARS-CoV-2 homology structures, doxycycline ranked in the group of compounds with the highest binding affinity to 3CLpro (Nsp5), the main protease in SARS-CoV-2 which is essential in the life-cycle of the virus and a major target for therapy (12). In contrast, no possible binding for major targets was observed for lopinavir and ritonavir, and no clear target of action was found for chloroquine phosphate. The homology structures of SARS-CoV-2 in this study were built on the known structures for SARS-CoV. Of note, 3CLpro is highly conserved between SARS-CoV and SARS-CoV-2 (12). Doxycycline may therefore have direct antiviral effects against SARS-CoV-2.

3. Targeted anti-inflammatory and tissue-protecting effects. Several studies are under way to target hyperinflammation in severe COVID-19 (13). Doxycycline exhibits significant clinically beneficial anti-inflammatory effects in various human diseases, including cystic fibrosis (14), atherosclerosis (15), lung fibrosis (16), sarcoidosis (17), and infection-induced hyperinflammation (18, 19). Doxycycline inhibits mitogen-activated protein kinase (MAPK) and Smad pathways (20), has potent antioxidant properties (21), and it also potently antagonises inflammatory metalloproteinases such as MMP9 that are implicated in lung injury, including SARS and ARDS (22).

4. Safety. Because possible beneficial effects of reducing inflammation in COVID-19 should be carefully weighed up against the potential for deleterious impairment of anti-microbial immunity" (23), doxycycline may be a safer choice than steroids or selective, highly potent immunosuppressive drugs. From a general drug perspective, a large safety database exists for doxycycline which was introduced to market in 1967. The incidence of adverse events (AE) is very low indeed (24), and the standard therapeutic dose of 200mg is also safe in patients with severely impaired renal function (25). Unlike Azithromycin and hydroxychloroquine, which can prolong the QT interval and, thereby come with a slight risk of torsade de pointe, especially in patients with cardiovascular co-morbidity and/or electrolyte imbalance. It is very often used together with a combination with chronic drugs, and also with antibiotics. Overall, the benefit:risk of doxycycline in COVID-19 patients has a high probability of being positive.

5. Pharmacokinetics (PK). Doxycycline's PK properties, including its almost complete bioavailability after oral dosing, short time required to achieve effective blood levels, half-life of 12-25h, strong tissue penetration, including respiratory tissues and sputum, and its reach into the intracellular space, match very well with the intended use of doxycycline in COVID-19 patients. Of note, the 200 mg per day dose of oral doxycycline allows for a concentration of doxycycline in the sputum that is capable of neutralising MMP-9 activity (26).

6. Cost and availability. In the UK, a 14-days treatment course of 200mg doxycycline costs £ 6.50. Doxycycline is produced by many companies, and as a drug that is listed on the WHO 2019 core list of essential medicines (27), so there should be sufficient stocked drug available in case it became a standard treatment for preventing severe COVID-19.

II. Study Design

Design: Parallel group, controlled (against standard of care, SoC), randomized (2:1 active:placebo), open-label trial design, including a screening period (0-1 days), a 14-days treatment period, and a 14-days follow up by telephone.

Patient population: Adult symptomatic patients, with a proven diagnosis of COVID-19 who have been admitted to hospital within the last 24h and who are at risk to develop severe COVID-19.

Inclusion criteria:

- Able to give informed consent
- Male or female, age ≥40 and <90 years
- Typical symptoms of COVUD-19 infection: new onset of or exacerbation of cough due to chronic respiratory illness, dyspnea, Increased body temperature (forehead T° >37.6°C; oral T°>38°C; axillary >37.5°C)
- SARS-CoV-2 infection demonstrated by PCR
- Admission to hospital within 10 days of onset of symptoms

Exclusion criteria: Hypersensitivity to doxycycline; myasthenia gravis; pregnancy; hepatic failure (CHILD-Pugh score C); unable to give informed consent.

Stratification: Assuming that the benefits may be different depending on prior co-morbidities, approximately equal numbers of patients will be recruited into the following strata (approx. 110 per group): no relevant prior illness; pre-existing lung conditions (ILD, COPD, bronchiectasis, asthma); and other relevant non-respiratory comorbidities (e.g. diabetes, heart disease, uncontrolled hypertension, cancer).

Treatment: Intention to treat with 200 mg of oral doxycycline per day (100 mg BID) for fourteen days on top of the standard of care (SOC), regardless of ability to complete the treatment course or subsequent use of rescue medication.

Follow-up: patients discharged before the 14-day period of expected follow up will be contacted by telephone 2 days after discharge (to ensure they are well and convalescing well) and on day 14 (by telephone).

Study endpoints:

- *Primary endpoint:* Need for transfer to ICU within 14 days of admission, as judged by the attending physicians

- Secondary endpoints: death, mechanical ventilation, time to discharge, recovery from symptoms, resolution of fever, prolonged hospital stay (>7days), supplemental oxygen required

Statistical considerations:

Question of scientific interest
The question of scientific interest that this trial is designed to assess is whether treatment with doxycycline reduces the need (indication) for ICU care in newly hospitalized patients with symptomatic COVID-19

Sample size justification
It is assumed that 25% of patients newly admitted to hospital with COVID-19 will require ICU care within 14 days of admission (28), and that the addition of 200mg doxycycline per day to standard of care will reduce the need for ICU transfer by 50%, a risk ratio of 0.500. Loss to follow up is assumed to be no more than 5%. A 2:1 randomisation ratio (Doxycycline plus SOC : SOC) will be used.

We require 80% power using a one-sided alpha of 2.5%.

The sample size required for a conventional trial, with no interim analyses, looking at the difference in the rate of transfer to ICU is 220 in the doxycycline group and 110 in the SOC group, a total of 330. Allowing for a 5% dropout rate, the corresponding figures are 231, 116 and 347.

Interim analyses and stopping rules
Interim analyses allowing the possibility of stopping for both success and futility shall be carried out once the status of 50% and 75% of participants will be known. In addition, a futility-only interim may occur once the status of 25% of participants will be known. A binding O'Brien-Fleming-like alpha spending function will be used to define the success boundary and a Pockock-like beta spending function will define the futility boundary.

The design characteristics of this design are summarised in the table below.

Operating characteristics of the chosen design				
	Interim 1	***Interim 2***	***Interim 3***	***Final***
Information rate	25.0%	50.0%	75.0%	100.0%
Total sample size*	110	220	329	439
Cumulative alpha spent	0.0000	0.0015	0.0092	0.0220
Cumulative power	0.000	0.250	0.649	0.800
One-sided local significance level	0	0.00153	0.00866	0.0189
Efficacy boundary (Z-value scale)	NA	2.963	2.380	2.078
Efficacy boundary (approximate treatment effect scale)	NA	-0.151	-0.107	-0.083
Futility boundary (Z-value scale)	0.153	0.933	1.531	NA
Futility boundary (approximate treatment effect scale)	-0.013	-0.055	-0.072	NA
Overall exit probability (under H1)	0.071	0.302	0.441	NA
Exit probability for efficacy (under H1)	0.000	0.250	0.399	0.151
Exit probability for futility (under H1)	0.071	0.053	0.042	NA
Overall exit probability (under H0)	0.561	0.287	0.112	NA

Exit probability for efficacy (under H0)<br>Exit probability for futility (under H0)	0.000<br>0.561	0.002<br>0.286	0.007<br>0.104	0.008<br>NA
*: Evaluable patients, without allowance for drop outs				

Specifically, the (approximate) values of observed treatment effect that would trigger a recommendation to stop the study at each analysis are given in the table below.

Approximate boundary values on the treatment effect scale		
Analysis	***Futility***	***Success***
Interim 1	-1.3%	
Interim 2	-5.5%	-15.1%
Interim 3	-7.2%	-10.7%
Final		-8.3%

For example, at the second interim, if the observed treatment effect is greater than -5.5%, the study will stop for futility. If it is less than -15.1%, it will stop for success. Otherwise, it will continue.

The maximum number of participants required using this design is 439, an increase of 109, before allowing for dropouts, compared to the conventional design. However, the expected sample size is reduced to 300.4 when doxycycline has the desired effect on ICU transfer and 178.9 when it has no effect.

It is unlikely that interims will occur precisely at the information fractions given above. Consequently, boundary values for subsequent interims were to be adjusted to ensure that the operating characteristics of the design are preserved. The adjusted boundary values for future interims would be documented in writing in the report that summarises the results of each interim analysis.

Some other properties of the design are summarised in the graphs below.

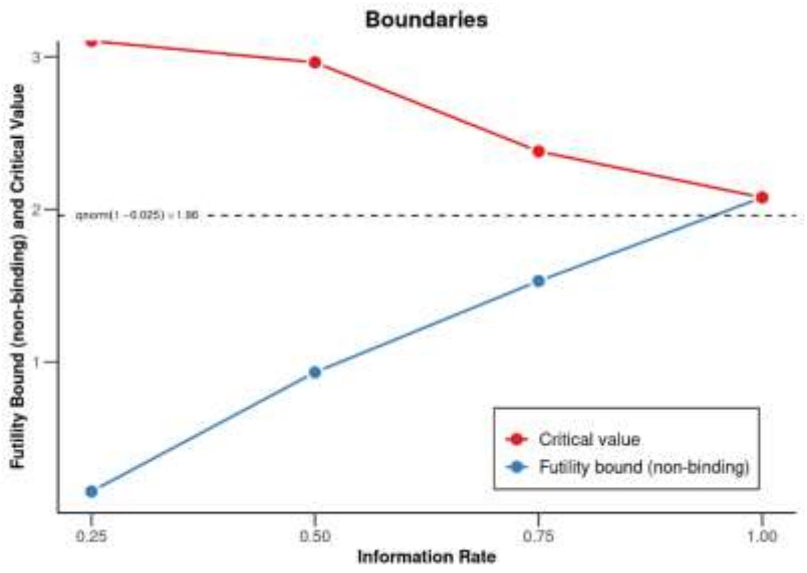

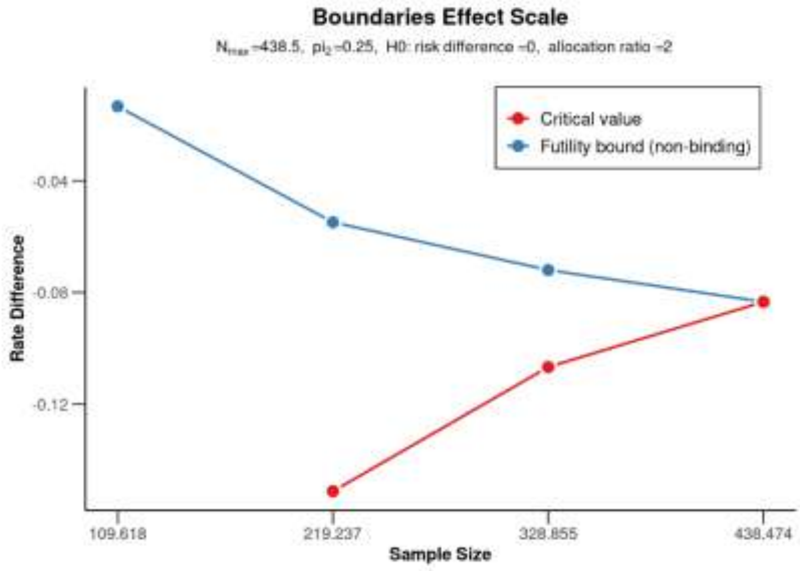

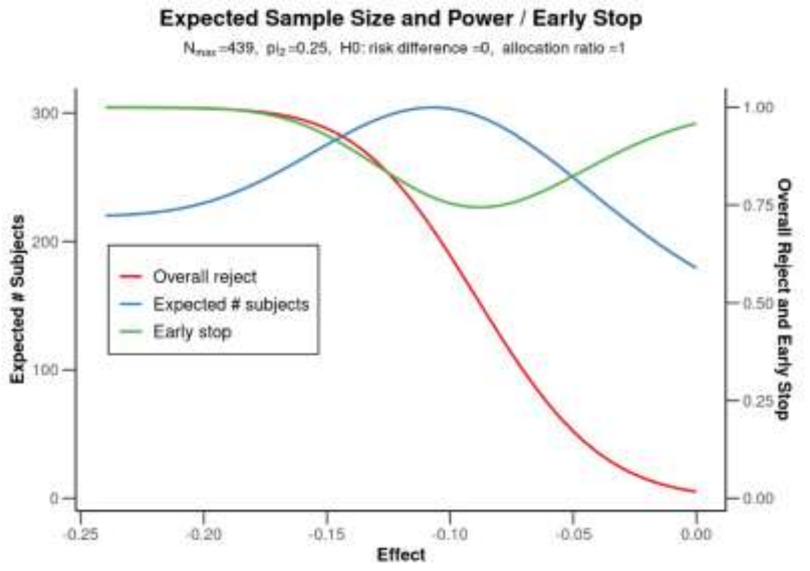

Data collection and analysis:

All data (including randomization, stratification, patient screening, treatment and outcomes data) will be collected by the attending physicians using an electronic case report form (eCRF) app for online entry. The eCRF app (which was especially developed for this trial) is hosted on a secure Amazon server that can be accessed by investigators via a user-specific username and password. The app consists of 5 pages: screening, randomization, treatment, adverse events, and outcome. Instructions for its use (see online supplement) will be available to all participants, and several online training sessions will be held, including completion of mock patient data. All users will need to be fully proficient before getting permission to enter real patient data. Specific instructions will be issued for when the data were to be placed within each of the five pages.

III. References

1) https://www.ecdc.europa.eu/sites/default/files/documents/RRA-seventh-update-Outbreak-of-coronavirus-disease-COVID-19.pdf
2) Baird, J. K. 2005. Effectiveness of antimalarial drugs. N. Engl. J. Med. 352:1565-1577.
3) Geisler WM, Uniyal A, Lee JY, et al. Azithromycin versus Doxycycline for Urogenital Chlamydia trachomatis Infection. N Engl J Med. 2015; 373:2512-21.
4) https://bestpractice.bmj.com/topics/en-gb/1604
5) Rothan HA1, Mohamed Z, Paydar M, et al. Inhibitory effect of doxycycline against dengue virus replication in vitro. Arch Virol. 2014 Apr;159(4):711-8. doi: 10.1007/s00705-013-1880-7. Epub 2013 Oct 19
6) Fredeking TM, Zavala-Castro JE, González-Martínez P, et al. Dengue Patients Treated with Doxycycline Showed Lower Mortality Associated to a Reduction in IL-6 and TNF Levels. Recent Pat Antiinfect Drug Discov. 2015;10(1):51-8.
7) Bhattacharjee B, Bhattacharya S, Sardar B, et al. Dengue and doxycycline-experience in a tertiary care hospital in eastern India. J Pharmacol Ther Res 2018; 2:14-17.
8) Rothan HA, Bahrani H, Mohamed Z, et al. A combination of doxycycline and ribavirin alleviated chikungunya infection. PLoS One. 2015;10:e0126360
9) Wu ZC, Wang X, Wei JC, et al. Antiviral activity of doxycycline against vesicular stomatitis virus in vitro. FEMS Microbiol Lett. 2015;362(22). pii: fnv195.
10) Li Y, Wu Z, Liu K, et al. Doxycycline enhances adsorption and inhibits early-stage replication of porcine reproductive and respiratory syndrome virus in vitro. FEMS Microbiol Lett. 2017 Sep 15;364(17). doi: 10.1093/femsle/fnx170.
11) Yan B, Chua YX, Lim AYN, et al. COVID-19 and false-positive dengue serology in Singapore. Lancet Infect Dis 2020 Mar 4. pii: S1473-3099(20)30158-4.
12) Wu C, Liu Y, Yang Y, et al. Analysis of therapeutic targets for SARS-CoV-2 and discovery of potential drugs by computational methods. Acta Pharmaceutica Sinica B, 2020 (accepted for publication).
13) Mehta PM, McAuley DF, Brown M, et al. COVID-19: consider cytokine storm syndromes and immunosuppression. Lancet 2020. https://doi.org/10.1016/S0140-6736(20)30628-0.
14) Xu X, Abdalla T, Bratcher PE, et al. Doxycycline improves clinical outcomes during cystic fibrosis exacerbations. Eur Respir J. 2017; 49. pii: 1601102.
15) Abdul-Hussien H, Hanemaaijer R, Verheijen JH, et al. Doxycycline therapy for abdominal aneurysm: Improved proteolytic balance through reduced neutrophil content. J Vasc Surg. 2009; 49:741-9.
16) Parthasarathi Bhattacharyya, Saikat Nag, Sujan Bardhan, et al. The role of long-term doxycycline in patients of idiopathic pulmonary fibrosis: The results of an open prospective trial. Lung India. 2009; 26: 81–85.
17) Bachelez H, Senet P, Cadranel J, et al. The use of tetracyclines for the treatment of sarcoidosis. Arch Dermatol. 2001 Jan;137(1):69-73.
18) Basheer A, Padhi S, Boopathy V, et al. Hemophagocytic Lymphohistiocytosis: an Unusual Complication of Orientia tsutsugamushi Disease (Scrub Typhus). Mediterr J Hematol Infect Dis. 2015; 7(1): e2015008.
19) Mangulabnan JL, Ogbac F. Effects of doxycycline on lowering IL-6 and TNF among patients with dengue haemorrhagic fever: a meta-analysis. 27th ESCMID. 2017.
20) Kim HS, Luo L, Pflugfelder SC, et al. Doxycycline inhibits TGF-β1-induced MMP-9 via Smad and MAPK pathways in human corneal epithelial cells. Invest Ophthalmol Vis Sci 2005; 46: 840–848
21) Clemens DL, Duryee MJ, Sarmiento C, et al. Novel Antioxidant Properties of Doxycycline. Int J Mol Sci. 2018;19(12). pii: E4078.
22) Hsu AT, Barrett CD, DeBusk GM, et al. Kinetics and Role of Plasma Matrix Metalloproteinase-9 Expression in Acute Lung Injury and the Acute Respiratory Distress Syndrome. Shock. 2015; 44:128-36.
23) Ritchie AJ, Singanayagam A. Lancet 2020. https://doi.org/10.1016/S0140-6736(20)30691-7
24) Smith K, Leyden JJ. Safety of doxycycline and minocycline: a systematic review. Clin Ther. 2005; 27:1329-42.
25) Stenbaek O, Myhre E, Berdal BP. Doxycycline (Vibramycin) in renal failure [Article in Norwegian language]. Tidsskr Nor Laegeforen, 1974 Dec 10;94(34-36):2388-9.
26) Beringer PM, Owens H, Nguyen A, et al. Pharmacokinetics of doxycycline in adults with cystic fibrosis. Antimicrob Agents Chemother 2012; 56: 70–74.
27) https://www.who.int/medicines/publications/essentialmedicines/en/
28) Wang D, Hu B, Hu C, Zhu F, Liu X et al. Clinical Characteristics of 138 Hospitalized Patients With 2019 Novel Coronavirus-Infected Pneumonia in Wuhan. JAMA 2020;323(11):1061-1069.
