# Supplementary material for: Doxycycline for the prevention of progression of COVID-19 to severe disease requiring intensive care unit (ICU) admission: A randomized, controlled, open-label, parallel group trial (DOXPREVENT.ICU)
Source: PLoS One. 2023 Jan 23;18(1):e0280745. doi: 10.1371/journal.pone.0280745 (PMC9870104; doi:10.1371/journal.pone.0280745)
Supplement: S2 File — (PDF) [file pone.0280745.s004.pdf]

Sample size calculation

(A) Original sample size calculation (per protocol)

Sample size justification

We assumed 25% of patients newly admitted to hospital with COVID-19 will require ICU care within 14 days of admission, and that the addition of 200mg doxycycline qd to standard of care will reduce the need for ICU transfer by 50%, a risk ratio of 0.500. Loss to follow up was assumed to be no more than 5%. A 2:1 randomisation ratio (Doxycycline plus SoC : SoC) was to be used.

We required 80% power using a one-sided alpha of 2.5%.

The sample size required for a conventional trial, with no interim analyses, looking at the difference in the rate of transfer to ICU was 220 in the doxycycline group and 110 in the SoC group, a total of 330. Allowing for a 5% dropout rate, the corresponding figures are 231, 116 and 347.

Interim analyses and stopping rules

Interim analyses allowing the possibility of stopping for both success and futility was to occur once the status of 50% and 75% of participants was known. In addition, a futility-only interim was to occur once the status of 25% of participants was known. A binding O'Brien-Fleming-like alpha spending function will be used to define the success boundary and a Pockock-like beta spending function will define the futility boundary.

The design characteristics of this design are summarised in the table below.

Operating characteristics of the chosen design				
	Interim 1	***Interim 2***	***Interim 3***	***Final***
Information rate	25.0%	50.0%	75.0%	100.0%
Total sample size*	110	220	329	439
Cumulative alpha spent	0.0000	0.0015	0.0092	0.0220
Cumulative power	0.000	0.250	0.649	0.800
One-sided local significance level	0	0.00153	0.00866	0.0189
Efficacy boundary (Z-value scale)	NA	2.963	2.380	2.078
Efficacy boundary (approximate treatment effect scale)	NA	-0.151	-0.107	-0.083
Futility boundary (Z-value scale)	0.153	0.933	1.531	NA
Futility boundary (approximate treatment effect scale)	-0.013	-0.055	-0.072	NA
Overall exit probability (under H1)	0.071	0.302	0.441	NA
Exit probability for efficacy (under H1)	0.000	0.250	0.399	0.151
Exit probability for futility (under H1)	0.071	0.053	0.042	NA
Overall exit probability (under H0)	0.561	0.287	0.112	NA
Exit probability for efficacy (under H0)	0.000	0.002	0.007	0.008
Exit probability for futility (under H0)	0.561	0.286	0.104	NA
*: Evaluable patients, without allowance for drop outs				

Specifically, the (approximate) values of observed treatment effect that would trigger a recommendation to stop the study at each analysis are given in the table below.

Approximate boundary values on the treatment effect scale		
Analysis	***Futility***	***Success***
Interim 1	-1.3%	
Interim 2	-5.5%	-15.1%
Interim 3	-7.2%	-10.7%
Final		-8.3%

For example, at the second interim, if the observed treatment effect is greater than -5.5%, the study will stop for futility. If it is less than -15.1%, it will stop for success. Otherwise, it will continue.

The maximum number of participants required using this design is 439, an increase of 109, before allowing for dropouts, compared to the conventional design. However, the expected sample size is reduced to 300.4 when doxycycline has the desired effect on ICU transfer and 178.9 when it has no effect.

It was thought unlikely that interims will occur precisely at the information fractions given above. Consequently, boundary values for subsequent interims were to be adjusted to ensure that the operating characteristics of the design are preserved. The adjusted boundary values for future interims would be documented in writing in the report that summarises the results of each interim analysis.

Some other properties of the design are summarised in the graphs below.

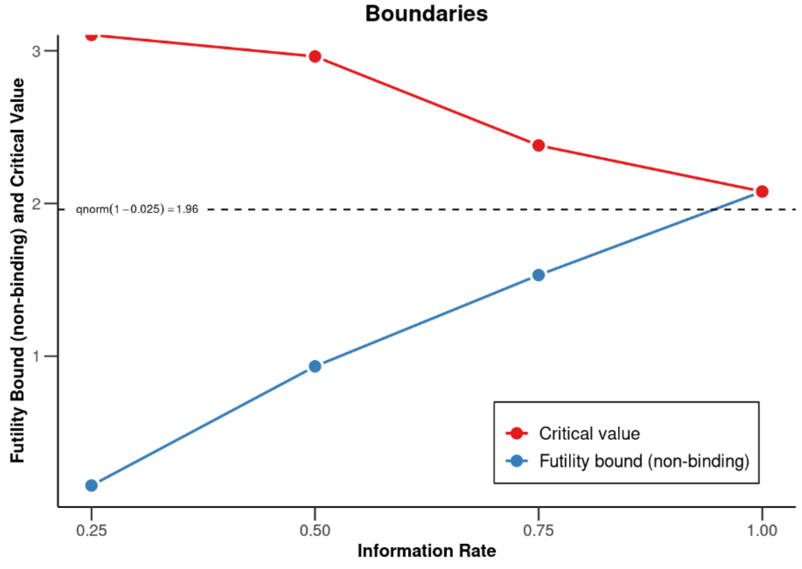

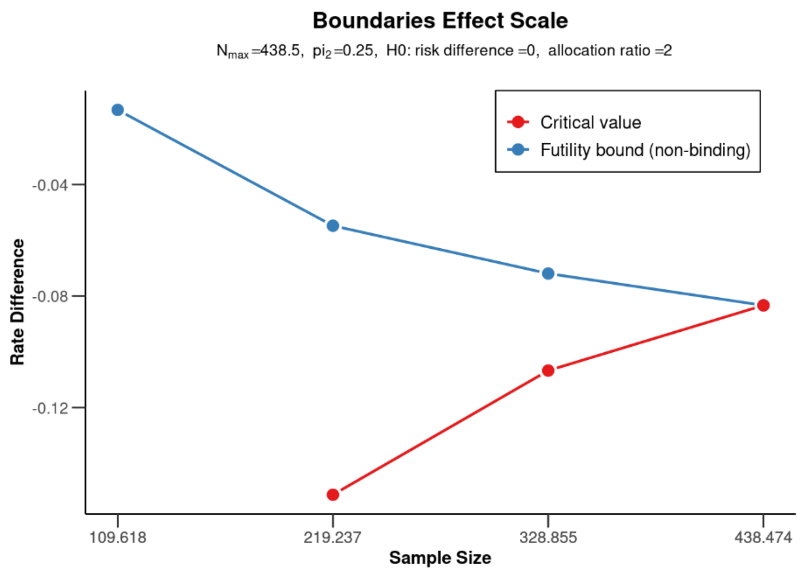

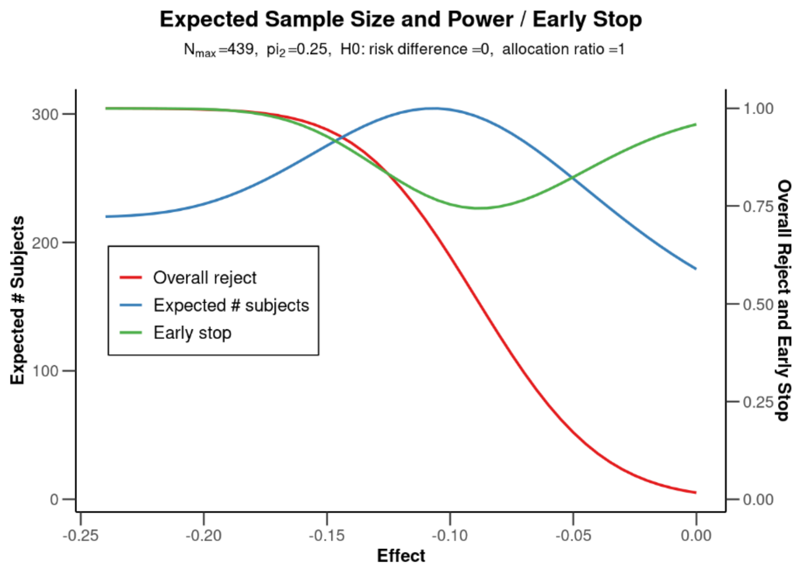

(B) Actual sample size calculation

Due to an error in programming, the study app was programmed for 1:1 randomisation.

We assumed that 25% of patients newly admitted to hospital with COVID-19 will require ICU care within 14 days of admission. The addition of 100mg doxycycline BID to standard of care will reduce the need for ICU transfer within by 50%, a risk ratio of 0.500. Loss to follow up will be no more than 5%. A 1:1 randomisation ratio (SoC+Doxy: SoC) will be used. Loss to follow up was assumed to be ≤5%.

We required 80% power using a one-sided alpha of 2.5%. The sample size required for a conventional trial, with no interim analyses, looking at the difference in the rate of transfer to ICU was 152 in the SoC+doxy arm and 152 in the SoC arm, a total of 304. Allowing for a 5% dropout rate, the corresponding figures were 160, 160 and 320. As we had already exceeded this number of evaluable subjects, no additional calculations to implement a group sequential approach were required.
