# Supplementary material for: Doxycycline for the prevention of progression of COVID-19 to severe disease requiring intensive care unit (ICU) admission: A randomized, controlled, open-label, parallel group trial (DOXPREVENT.ICU)
Source: PLoS One. 2023 Jan 23;18(1):e0280745. doi: 10.1371/journal.pone.0280745 (PMC9870104; doi:10.1371/journal.pone.0280745)
Supplement: S3 File — (PDF) [file pone.0280745.s005.pdf]

Detailed material and methods of in vitro studies

Cell culture. Vero-E6 cells (American Type Culture Collection, ATCC, Virginia, USA), A549 cells (human adenocarcinomic alveolar basal epithelial cells; American Type Culture Collection, ATCC, Virginia, USA), Caco-2 cells (human colorectal adenocarcinoma cell line, ATCC, Virginia, USA) and MDA-MB-231 cells (triple-negative human breast adenocarcinoma; DSMZ-German Collection of Microorganisms and Cell Cultures GmbH, Braunschweig, Germany) were cultivated at 37°C in a humidified incubator with atmospheric oxygen concentrations (21 %) and 5 % CO2. Cells were maintained in Dulbecco's Modified Eagle's Medium (DMEM) high glucose containing 10% fetal bovine serum (FBS), 100 U/mL penicillin-streptomycin and NEAA (culture medium). Cells were routinely passaged when reaching a confluence of 80 - 90%.

Isolation and expansion of SARS-CoV-2 clinical isolates. Caco-2 cells cultivated in "virus isolation medium" (DMEM, 2% FBS, 100 U/mL penicillin-streptomycin, NEAA, 0.5 µg/mL gentamicin, and 0.25 µg/mL amphotericin B) were challenged for 2 h with a clinical isolate of the B.1.177 (EU1) lineage (GISAID EPI ISL: 3233461) obtained from a nasopharyngeal swab of a COVID-19 patient. Subsequently, the virus isolation medium was replaced with regular culture medium, and three days post infection supernatant was collected and passaged onto Vero-E6 cells (ATCC) in virus isolation medium. After three additional days, cell culture supernatants were harvested and stored at -80°C. Further expansion of viruses was performed in "virus expansion medium" (DMEM containing 5% FBS, 100 U/mL penicillin-streptomycin, NEAA). VOCs Alpha (B.1.1.7; GISAID EPI ISL: 3233462), Beta (B.1.351; (GISAID EPI ISL: 1752394), Gamma (P.1 / B.1.1.28.1; (GISAID EPI ISL: 2095178)) and Delta (B.1.617.2; (GISAID EPI ISL: 2772700)) were kindly provided by the Bavarian Landesamt für Gesundheit und Lebensmittelsicherheit (LGL). VOC Alpha + E484K (B.1.1.7 + E484K; GISAID EPI ISL: 2772697) was obtained from the Institute for Virology, Innsbruck, Austria. All VOCs were expanded in expansion medium on Vero-E6 cells. Virus stocks were characterized by RT-qPCR, as reported previously (1). In parallel, for expanded stocks of SARS-CoV-2 near full-length genome sequences were obtained following the ARTIC network nCoV-2019 sequencing protocol v2 (2) as described previously (3).

Infection of A549-hACE2 cells and MDA-MB-231-hACE2 cells with SARS-CoV-2 – drug screening viability assay. A549-hACE2 cells (7.5 x 103 cells per well) and MDA-MB-231-hACE2 cells (1.0 x 104 cells per well) were plated in a 384-well white well plate (Corning) in "virus infection medium" (DMEM, 2% FBS, 100 U/mL penicillin-streptomycin, NEAA). Target cells were treated with either medium or a serial dilution of remdesivir (Adooq Biosciences), doxycycline (Sigma-Aldrich) or tetracycline (Sigma-Aldrich) six hours before infection. Subsequently, cells were challenged with the indicated SARS-CoV-2 clinical isolates. The volume of inoculum was chosen according to titration on A549-hACE2 cells and MDA-MB-231-hACE2 cells aiming to reach ~10% viability of untreated target cells at the time point of harvest. 48 h (MDA-MB-231-hACE2 cells) or 72 h (A549-hACE2 cells) after infection, analysis of virus-induced killing was performed by measurement of viability of target cells using the CellTiter-Glo 2.0 reagent (Promega). Cells were treated according to the manufacturer's instructions. In brief, 10 µl CellTiter-Glo 2.0 reagent was added to each well, incubated for 10 min in the dark at room temperature and luminescence was recorded using the Infinite F200 microplate reader (Tecan). Viability of cells was calculated by normalization of readings for infected cells relative to those for untreated control cells. Curve fitting and EC50 calculation was done using GraphPad Prism (non-linear regression).

References

1. M. Muenchhoff et al., Multicentre comparison of quantitative PCR-based assays to detect SARS-CoV-2, Germany, March 2020. Euro Surveill. 25, 2001057 (2020).
2. J. R. Tyson et al., Improvements to the ARTIC multiplex PCR method for SARS-CoV-2 genome sequencing using nanopore. bioRxiv, 2020.2009.2004.283077 (2020).
3. T. Weinberger et al., Prospective Longitudinal Serosurvey of Health Care Workers in the First Wave of the SARS-CoV-2 Pandemic in a Quaternary Care Hospital in Munich, Germany. Clin. Infect. Dis., (2021).
