# Supplementary material for: Doxycycline for the prevention of progression of COVID-19 to severe disease requiring intensive care unit (ICU) admission: A randomized, controlled, open-label, parallel group trial (DOXPREVENT.ICU)
Source: PLoS One. 2023 Jan 23;18(1):e0280745. doi: 10.1371/journal.pone.0280745 (PMC9870104; doi:10.1371/journal.pone.0280745)
Supplement: Attachment — Submitted filename: Response to Reviewers.docx [file pone.0280745.s007.docx]

**Response to Reviewers**

**PONE-D-22-10587**

Doxycycline for the prevention of progression of COVID-19 to severe disease requiring intensive care unit (ICU) admission: a randomized, controlled, open-label, parallel group trial (DOXPREVENT.ICU)

**I. Dear Editor and reviewers,**

Thank you for your careful review of our manuscript and your valuable suggestions on how to improve it.

We are happy to provide below our point-by-point responses to each of the comments/critique raised by the two reviewers. In the following response, text edits in the revised manuscript (changes to the original text) are marked-up by using **bold text on yellow background**.

We have uploaded both a “Revised Manuscript with Track Changes” that highlights changes made to the original version, and an unmarked version (“Manuscript”).

Finally, as requested by the academic editor, we have also addressed the additional style requirements, including those for captions of submitted files.

**II. Responses to reviewers’ comments on general aspects of the manuscript.**

**1) Is the manuscript technically sound, and do the data support the conclusions?**

*Response:* We thank both reviewers for agreeing that our manuscript is technically sound and that the data support the conclusions.

**2) Has the statistical analysis been performed appropriately and rigorously?**

*Response:* Reviewer #2 raised a couple of points in relation to the statistical analysis. We will address these points below (in the section « 5. Response to reviewers’ comments on specific aspects of the manuscript »).

**3) Have the authors made all data underlying the findings in their manuscript fully available?**

*Response:* We thank both reviewers for appreciating our effort to make all data underlying our findings available to the public.

**4) Is the manuscript presented in an intelligible fashion and written in standard English?**

*Response:* Thank you for appreciating the clarity and writing style of our manuscript.

**III. Point-by-point responses to reviewers’ comments on specific aspects of the manuscript.**

**Points raised by Reviewer #1**

**1) PRINCIPLE, UK platform found that doxycycline was not associated with reduced hospital admission or death when used in the community in patients with suspected early COVID-19.**

*Response:* The main differences between the trial reported in the manuscript under consideration and that reported by the PRINCIPLE consortium, to which three of us contributed as co-authors, relate to the patient population enrolled in the respective trials. While PRINCIPLE was a community-based trial for all-comers from age 18 onwards, therefore including mostly people with mild COVID-19, our trial was designed to test the effect of doxycycline in patients who were severe enough to require hospital admission. Thus, the patients in the current trial had more advanced disease stage COVID-19 and were, therefore, at a much higher risk for severe outcome. Of note, during the 2nd wave of the pandemic in India, hospitals were at their capacity and stretched to their limit and only severely ill patients could be admitted. In fact, the situation was so dramatic that many COVID-19 patients died outside of these hospitals before they could be admitted). The differences in population between our trial and PRINCIPLE are evident from the very different (almost 20-fold different) death rate between the two trials, i.e. 0.6% death rate in PRINCIPLE versus 10.1% death rate in our trial. In conclusion, we argue that these two trials cannot be directly compared.

To emphasize this point more clearly in the manuscript, we have revised the text of the manuscript as follows (new sentence shown in **bold**):

*Section: “Discussion”; 5^th^ paragraph:* «The UK platform trial, PRINCIPLE, to which some members of our study team contributed (SG, JK, RD), found that doxycycline was not associated with reduced hospital admissions or death when used in the community in patients with suspected early COVID-19.^23^ **Of note, the population of this trial was much more severe than that studied in PRINCIPLE, a community-based platform trial. While most patients enrolled in PRINCIPLE had mild disease, all patients in this trial were severe enough to warrant hospital admission.** This contrast in benefits of doxycycline **between the PRINCIPLE** **and** the current study of advanced hospital stages of COVID-19 further supports the notion that doxycycline has no direct antiviral activity against SARS-CoV-2. Of note, dependency on the stage of disease is also observed with monoclonal antibodies against the SARS-CoV-2 spike protein which act directly as anti-viral agents and are effective during early disease.^20^»

**2) No difference was seen in the secondary outcome measure of death between the two treatment arms in this study.**

*Response:* We have discussed this in the manuscript (Discussion, 3^rd^ paragraph), based on the results described in the section “Key secondary outcomes”. Due to space constraints and the fact that it is a secondary outcome measure, we kept this part of the discussion brief. However, if the reviewer insists and the editor agrees, we would be happy to expand in a further revision. In our view, two main aspects of this trial may explain why no statistically significant difference in death rate was observed between the two treatment arms:

1. *Our prospective randomised clinical trial was not powered to see differences in death rate between the two study arms:*

- A retrospective power calculation, based on the observed death rate of 10% in the SoC group, shows that a sample size of 868 patients (i.e. more than 2x the number of patients included in our trial) would have been required to observe (with alpha 0.05 and power 80%) a (quite dramatic) 50% reduction of the death rate. In order to observe a lesser, albeit still highly relevant 25% reduction of the death rate, a sample size of 4008 patients (i.e. >10x the number of patients in our trial) would have been required. A trial of that size was simply beyond our means.

2. *Differences in ICU admission rates for patients of the two study arms who reached the primary outcome of “need for ICU admission”:*

- A notable result of our study was that all but 1 of the 31 patients, judged by their doctors to require ICU treatment but could not be admitted to ICU, died (96.8%), while only 1 of 5 patients (9 of 46, 19.6%) admitted to ICU died. Thus, those patients in our trial whose COVID-19 deteriorated to the extent that they reached the primary outcome “need for ICU treatment” and could be admitted to ICU stood a much better chance of survival than those who reached the primary outcome but were not admitted to ICU for reasons that we explain in the paper.
- By chance (for unknown reasons), only 46% (12 of 26) of patients in the Doxycyline arm who reached the primary outcome were actually admitted to ICU, compared to 67% (34 of 51) of patients in the SoC arm. This bias in ICU admission rates, for which we have no explanation, may have confounded the secondary outcome of death in our study.

**3) Results presented at the American College of Allergy, Asthma & Immunology (ACAAI) 2021 Annual Scientific Meeting are similar (to results of the PRINCIPLE trial). Use of doxycycline is not associated with improved mortality rate in patients with COVID-19 pneumonia. How to explain these results?**

*Response:* The study presented at the American College of Allergy, Asthma & Immunology (ACAAI) 2021 Annual Scientific Meeting was a retrospective chart study in 110 patients with COVID-19 pneumonia. The limited information provided in the brief study abstract does not allow us to assess the patient population or other relevant aspects of the study. It is, therefore, not possible to conduct a fair comparison between this the ACAAI study and our prospective randomised controlled trial.

**4) Authors mentioned that doxycycline has no *in vitro* activity against SARS-CoV-2. This is in contrast with findings from other authors (e.g. Gendrot).**

*Response:* Thank you for raising this difference between our own *in vitro* study and that of Gendrot et al. For additional clarity, we have added text in the methods section of the main manuscript, adding an appropriate reference, and have expanded the discussion on previous *in vitro* studies, also adding three references.

(Both the numbering of references and the list of references were updated accordingly).

Please note the following edits in the revised manuscript:

*Section: “Methods”; subsection “In vitro studies”, 1^st^ sentence:* «To test and compare the effects of doxycycline, tetracycline and remdesivir on SARS-CoV-2 infection of ACE-2-overexpressing lung-derived cell line **A549-hACE2 and breast cancer cell line MDA-MB-231-hACE2**, we carried out a **standardized, luminometric** viability assay **following drug titration and exposure to six SARS-CoV-2 variants, in principle as reported.^16^**»

*Section: “Discussion”, 4^th^ paragraph, edited sentences 4 and new sentences 5-7:* «**Our** *in vitro* studies showed that **neither** doxycycline **nor tetracycline** had **an inhibitory effect on different SARS-CoV-2 variants, including five variants of concern evaluated on two established ACE-2-overexpressing human cell lines (*PMID: 35090165*) (Suppl. Figure 1). As an antiviral positive control, the clinical RNA-dependent RNA polymerase (RdRp) inhibitor remdesivir potently blocked SARS-CoV-2 replication and virus-induced cell death (Suppl. Figure 1).^16^ While studies by one laboratory in African green monkey-derived Vero-E6 cells suggested an antiviral effect of doxycycline *in vitro*,^20, 21^_,_ a report by another group has challenged this conclusion.^22^ Potentially, the non-human origin of Vero-E6 cells may have been a confounder in the evaluation of anti-SARS-CoV-2-specific effects of doxycycline.**»

**Points raised by Reviewer #2:**

**1) Mortality is not clearly defined. Is it measured at discharge or in follow-up? A survival analysis may be more suitable.**

*Response:* Thank you for seeking more clarification.  Survival analysis is normally conducted on data for which the period of observation is long. Given the relatively short duration over which the primary outcome was assessed (14 days) we did not, and still do not, think that a time-to-event analysis would add any useful insight.

We are grateful for the question about the time-point at which mortality was measured and have, therefore, edited the text as follows:

*Section: “Discussion”; subsection “Key secondary Outcomes”, New 2^nd^ sentence in 2^nd^ paragraph:* «**All deaths occurred in hospital. No deaths were recorded after discharge during the 14-day telephone follow-up period.**»

**2) Most secondary outcomes were not analyzed or presented in the results section.**
*Response:* In this paper we have focused on the primary outcome “need for ICU admission”. The secondary outcome data are available for independent analysis via the supplementary information. However, any analysts seeking to undertake this should be aware that data collection for some secondary endpoints was not as complete as we had hoped due to pressures caused by the pandemic wave that India was going through at the time.

**3) The results section was not clearly written. The primary analysis should focus on the comparison between two treatment groups. However, in “Primary Outcome”, primary findings were buried in subgroup analysis (Eg. Comorbidities, lung disease and etc). The subgroup analysis should be moved to another section.**

*Response:*

We agree with the reviewer that the clarity of this section can be improved. We have, therefore, edited the text in the “Primary Outcome” section as shown below.

We also agree with the reviewer that the focus of the primary analysis must be on the comparison between treatment groups, although we argue that we have actually built the section in this way. The first, introductory paragraph of the “Primary Outcome” section provides the relevant primary outcome data of the whole trial population and of the clinically important strata. As these are actual primary outcome data, we argue they should stay in this (“Primary Outcome”) section of the manuscript. On the other hand, to put them at the end of this section, which (in the actual version) ends with the most exciting result of the trial, seems inappropriate.

Edited text as follows:

*Section: Results; subsection “Primary Outcome”, 1^st^ paragraph, first sentence:*

«Of the 387 patients included in the trial, 77 (19.9%) **reached the primary outcome, i.e., they** developed COVID-19 symptoms deemed by the attending physicians severe enough to require critical care in the ICU (Figure 3).»

*Section: Results; subsection “Primary Outcome”, 2^nd^ paragraph, a new, first sentence and a slightly modified, second sentence as follows:*

«**For comparison of the primary outcome between the two treatment arms in this trial we carried out three types of analysis: First,** **i**n the intention to treat analysis (ITT) of all 387 participants, 46 of 195 patients (23.6%) randomized to SoC **reached the primary outcome** compared to 31 of 192 patients (16.1%) randomized to SoC+Doxy (OR 0.617, 95% CI 0.369 to 1.027, p=0.063) (Figure 4).»

**4) In “Key secondary Outcomes”, ICU admission were mentioned again. This is confusing as ICU is not secondary but primary.**

*Response:* Thank you for giving us the opportunity to clarify. The primary endpoint of our trial is ***need*** for ICU admission. However, as explained in the manuscript, not all patients reaching this (primary) endpoint could actually be admitted to ICU, for reasons explained in the manuscript. Thus, actual ICU admission was included as a secondary endpoint and is discussed under key secondary outcomes in the manuscript. The reference of the primary outcome of ***need*** *for* ICU admission is to contextualise the secondary (but closely related) endpoint of actual admission.

**5) Table 1 present either mean with SD or median with range. How are the two groups compared?**

*Response:* We present means and ranges for continuous variables and n (%) for categorical ones.  We can amend to mean (SD) if the editor/reviewers consider this essential for acceptance, but we feel the current presentations provide an equivalent level of detail.  Interested readers have access to the raw data via the paper’s supplements if they are sufficiently motivated to derive alternative summaries of their own.

**6) Add p values to Table 1 and 2.**

*Response:* We respectfully disagree with the suggestion from reviewer 2 that p-values should be added to this table (and to table 2). Such an action is contrary to guidance from the CONSORT Consortium which states that "such significance tests assess the probability that observed baseline differences could have occurred by chance; however, we already know that any differences are caused by chance. Tests of baseline differences are not necessarily wrong, just illogical"

(for full guidance please see https://www.consort-statement.org/checklists/view/32--consort-2010/510-baseline-data, second para) See also deBoer et al (2015) Int J Behav Nutr Phys Act <https://doi.org/10.1186%2Fs12966-015-0162-z>

**7) Table 2 put n (%) in one cell.**

*Response:* Table 2 has been edited according to the reviewer’s suggestion (please see revised manuscript).

**8) Figure 2 a flow chart for outcome is not necessary and can be omitted.**

*Response:* We believe Reviewer #2 is actually referring to Figure 3, as Figure 1 is the CONSORT diagram which needs to be included in the manuscript as per guidance of the journal, and Figure 2 is showing enrollment into the trial. The flow chart in Figure 3 provides the reader with an overview of the main results. We agree that it could be omitted from the main manuscript text and propose (to the academic editor) that, if he so chooses, the current Figure 3 can be moved to the supplementary section.

**9) “The two treatment groups were matched” on Page 7 study participants and Page 8 2nd paragraph. The authors may change the statement to “two groups are not significantly different”. Matching has different meaning in statistics.**

*Response:* We thank the editor for this comment and have edited, accordingly, the text in the manuscript.

Please see the following edited text in the manuscript:

*Section: “Results”, subsection “Study participants”, 2^nd^ paragraph, third sentence, as follows:*

«**The incidence of various risk factors (RF) was similar in the two treatment groups** (Table 1).»

*Section: “Results”; subsection “Study treatment”, 2^nd^ paragraph, first sentence, as follows:*

«**Participants in the two treatment groups were similar with respect to** concomitant medication (Table 1), …»

**Final note to academic editor**

On review of our manuscript, we have deleted the following sentence from the manuscript as it is redundant:

Section: “Methods”, subsection “*Data collection and analysis*”, 2^nd^ paragraph, deleted the following (second) sentence «**~~Data could not be persisted on the app and, therefore, needed to be downloaded on every load, and new data needed to be uploaded before exit; thus, the app synchronized all changes in real time with a remote database in the cloud.~~**»

Thank You for considering our revised manuscript for publication in PLoS One.
