# Supplementary material for: Doxycycline for the prevention of progression of COVID-19 to severe disease requiring intensive care unit (ICU) admission: A randomized, controlled, open-label, parallel group trial (DOXPREVENT.ICU)
Source: PLoS One. 2023 Jan 23;18(1):e0280745. doi: 10.1371/journal.pone.0280745 (PMC9870104; doi:10.1371/journal.pone.0280745)
Supplement: Attachment — Submitted filename: 2nd Response to Reviewers_Dec 2022.docx [file pone.0280745.s008.docx]

**Response to Reviewer 2**

**PONE-D-22-10587**

Doxycycline for the prevention of progression of COVID-19 to severe disease requiring intensive care unit (ICU) admission: a randomized, controlled, open-label, parallel group trial (DOXPREVENT.ICU)

Dear Editor and reviewers,

We thank Reviewer #1 for his conclusion that all major and minor comments have been adequately addressed in our previously submitted revised manuscript.

Reviewer #2 raised the following point to which we will respond in the following.

**Points raised by Reviewer #2 (in response to submitted 1^st^ version of our revised manuscript):**

**1) For table 1, please follow the PLOS ONE guidelines**
(<https://www.equator-network.org/wp-content/uploads/2013/03/SAMPL-Guidelines-3-13-13.pdf>):

*Summarize data that are approximately normall distributed with means and standard deviations (SD). Use the form: mean (SD), not mean ± SD.*

*Summarize data that are not normally distributed with medians and interpercentile ranges, ranges, or both. Report the upper and lower boundaries of interpercentile ranges and the minimum and maximum values of ranges, not just the size of the range*

*Response:* As it is unusual, in our experience, to distinguish between normal and non-Normal data rather than continuous and discrete data, it would be helpful if Reviewer 2 could indicate which continuous variables they regard as “approximately normally distributed” and those which they do not.

We are also unsure about the comment of Reviewer 2 in relation to “interpercentile range” without specification of the percentiles that define the lower and upper boundaries of the range. Reporting interquartile ranges as well as minima, maxima and medians makes no sense for dichotomous and other categorical variables (which are by definition not Normal), such as gender, baseline PCR test results, the presence or absence of comorbidities and the use of concomitant medications.

In an effort to oblige Reviewer 2’s comment, we have made the following changes to the text of the manuscript on page 7, “Results”, first paragraph of “Study participants”, 3^rd^ and 4^th^ sentence, to include median, IQR, min and max for age and temperature:

“The **median** age (**IQR, min, max**) of participants was **58 (49 – 66, 40, 90)** years, with a 2:1 ratio of males to females. The **median** axillary body temperature (**IQR, min, max**) at study entry was 38.2°C (**38.0°C – 38.4°C, 37.8°C, 40.8°C**) in the SoC+Doxy group and **38.3° C (37.6°C – 38.3°C, 37.6°C, 40.6°C)** in the SoC group.”

In the revised Table 1, we summarise the following variables:

| Variable | Comment |
| --- | --- |
| Age | Continuous, but not Normal. We currently report mean and range. I’ve added the requested summaries in reviewer2-requests.pdf |
| Sex | Dichotomous. We currently report n and percentage. I suggest no change |
| Baseline PCR status | Dichotomous. We currently report n and percentage. I suggest no change |
| Body temp | Continuous. Is it Normal? Probably not. I’ve added the requested summaries in reviewer2-requests.pdf |
| Comorbidities | Dichotomous. We currently report n and percentage. I suggest no change |
| Con meds | Dichotomous. We currently report n and percentage. I suggest no change |

Table 2: Suggest no changes: all variables are dichotomous.

**Final note to academic editor**

Thank You for all your efforts with our manuscript and for considering this revised version for publication in PLoS One.
